# Core/Double-Sheath Composite Fibers from Poly(ethylene oxide), Poly(L-lactide) and Beeswax by Single-Spinneret Electrospinning

**DOI:** 10.3390/polym14225036

**Published:** 2022-11-21

**Authors:** Selin Kyuchyuk, Dilyana Paneva, Nevena Manolova, Iliya Rashkov, Daniela Karashanova, Nadya Markova

**Affiliations:** 1 Laboratory of Bioactive Polymers, Institute of Polymers, Bulgarian Academy of Sciences, Acad. G. Bonchev St., Bl. 103A, BG-1113 Sofia, Bulgaria; 2 Institute of Optical Materials and Technologies, Bulgarian Academy of Sciences, Akad. G. Bonchev St., Bl. 109, BG-1113 Sofia, Bulgaria; 3 Institute of Microbiology, Bulgarian Academy of Sciences, Akad. G. Bonchev St., Bl. 26, BG-1113 Sofia, Bulgaria

**Keywords:** electrospinning, nanofibrous composite, core/double-sheath fibers, core-shell fibers, beeswax, poly(L-lactide), poly(ethylene oxide), antimicrobial activity

## Abstract

The conventional approach for preparation of core-sheath fibers is coaxial electrospinning. Single-spinneret electrospinning of emulsions is a much less common method to obtain core-sheath fibers. Core-sheath structure may be generated by electrospinning of homogeneous blend solutions; however, reports on such cases are still scarce. Herein, the preparation of nanofibrous composites from poly(ethylene oxide) (PEO), poly(L-lactide) (PLA) and beeswax (BW) by single-spinneret electrospinning of their homogeneous blend solutions in chloroform is reported. The produced fibers had core/double-sheath structure with a PEO core, PLA inner sheath and BW outer sheath. This original fiber structure was evidenced by transmission electron microscopy, selective extraction of BW or PEO, and X-ray photoelectron spectroscopy. The PLA/BW double sheath led to hydrophobicity of the PEO/PLA/BW mats. The tensile tests revealed that PEO/PLA/BW mats had substantially improved mechanical behavior as compared to PEO, PLA and PEO/BW mats. PEO/PLA/BW mats can be used as drug carriers as evidenced by the one-pot incorporation of the model drug 5-nitro-8-hydroxyquinoline (NQ) into the fibrous materials. Microbiological tests showed that PEO/PLA/BW/NQ had antimicrobial activity. Therefore, the new materials are promising for wound healing applications.

## 1. Introduction

The knowledge gained during the last two decades about the preparation of polymer materials by electrospinning has proven that this technology enables the formation of fibers which might have a great variety of architectures [1,2,3,4]. An attractive class of electrospun fibers is the class of core-sheath fibers, due to the possibility of combining the properties of two polymers or a polymer and a low-molecular-weight substance [5,6,7]. To date, the following approaches for preparation of core-sheath fibers by electrospinning have been employed: coaxial electrospinning [8,9,10,11], emulsion electrospinning [12,13] and single-spinneret electrospinning in conjunction with simultaneous or subsequent chemical treatment [14,15]. An interesting approach for the preparation of core-sheath fibers is the single-spinneret electrospinning of homogeneous polymer blend solutions. The obtaining of core-sheath fibers by applying this approach is attributed to the occurrence of phase separation between the partners during the electrospinning process. The single-spinneret electrospinning of homogeneous polymer blend solutions is very attractive due to the fact that it does not require any of the following: the use of an auxiliary device for coaxial electrospinning to the electrospinning set-up the use of an emulsifier; or performance at a second stage of chemical treatment for formation of the fiber’s sheath. Until now, the preparation of fibers consisting of a core and a sheath by using this approach is known [16,17,18,19,20,21,22,23,24].

Biocompatible and biodegradable materials are of particular interest. Poly(ethylene oxide) (PEO) is a water-soluble polymer widely used in the medical and pharmaceutical practices [25] due to its non-toxicity and biocompatibility. In addition, owing to its non-ionogenic nature, fibers from PEO are prepared easily by electrospinning [26,27,28,29]. Poly(L-lactide) (PLA) is a biocompatible and (bio)degradable polymer and has become one of the most promising biobased polymers [30,31,32]. In addition, PLA has good mechanical strength. Beeswax (BW) is a water-insoluble natural product that is a complex mixture of low-molecular-weight substances: saturated hydrocarbons, fatty acids, fatty alcohols, and esters of fatty acids and fatty alcohols; the esters represent ca. 70% of BW’s composition [33]. It has been found that myricyl palmitate is the main ester in BW composition [34]. BW finds wide application in the food and pharmaceutical industry, as well as in cosmetics [35,36]. As can be seen, PEO, PLA and BW possess desirable and attractive properties that make them applicable in the fields of medicine, (agro)pharmacy, cosmetics, etc. For the preparation of core-sheath fibers by single-spinneret electrospinning of homogeneous blend solutions, in most cases polymers with widely differing properties have been combined. For example, PLA and BW are soluble in organic solvents, while PEO is a typical water-soluble polymer. In order to obtain fibers of intended design from these components by single-spinneret electrospinning, some significant challenges have to be overcome: (i) to find a common solvent for the components, and (ii) to find conditions inducing phase separation so that a well-defined core and sheath(s) can be observed.

The aim of the present study is to use our previous experience in the preparation of core-sheath-like fibers from PEO/BW [37] to obtain PEO/PLA/BW fibers with a similar architecture by single-spinneret electrospinning of their homogeneous blend solution at different ratios between the components, as well as to evaluate the possibility of incorporating of a low-molecular-weight bioactive substance for imparting antibacterial and antifungal properties to the new materials. Our choice of PLA has been made assuming that, in this way, fibrous materials with hydrophobic surfaces and with improved mechanical properties compared to PEO/BW mats may be obtained. Hydrophobicity may be achieved if the surface layer of the fibers consists of the hydrophobic PLA and BW. Studies of the design, structure and properties of core-sheath fibers prepared by single-spinneret electrospinning of homogeneous blend solutions are still scarce. In addition, no data have been found on methods for one-pot preparation of composite fibers with core/double-sheath structure. The new PEO/PLA/BW fibrous materials prepared by electrospinning were characterized by scanning and transmission electron microscopy. Further, the materials were studied with respect to the chemical composition of their surface, wettability, thermal behavior, crystallinity, as well as their mechanical behavior. The potential of PEO/PLA/BW mats to serve as carriers of bioactive substances was evaluated using the model drug 5-nitro-8-hydroxyquinoline (NQ). The effect of NQ-containing mats was studied in vitro against the pathogenic microorganisms *Staphylococcus aureus*, *Escherichia coli*, *Pseudomonas aeruginosa* and *Candida albicans*.

## 2. Materials and Methods

### 2.1. Materials

Poly(ethylene oxide) (PEO, Badimol-M^®^ Dimitrovgrad, Bulgaria) was used. Its viscosity-average molar mass (600,000 g/mol) was determined in distilled water at 30 °C by Ubbelohde viscometer using the equation: [η] = 1.25 × 10^−4^ × Mη^0.78^ [38]. Poly(L-lactide) (PLA) was also used (Ingeo™ Biopolymer 4032D, NatureWorks LLC—USA; M_w_ = 259,000 g/mol; M_w_/M_n_ = 1.94; as determined by size-exclusion chromatography using polystyrene standards). Beeswax with purity corresponding to the European Pharmacopoeia was bought from Chemax Pharma Ltd., Sofia, Bulgaria.; 5-Nitro-8-hydroxyquinoline (NQ) from Sigma-Aldrich (St. Louis, MO, USA); and chloroform, hexane, NaH_2_PO_4_ and KH_2_PO_4_ from Merck (Darmstadt, Germany). All of the above-mentioned chemicals were reagents for analytical applications and were used as received. *Staphylococcus aureus* strain 749 (*S. aureus*), *Escherichia coli* strain 3588 (*E. coli*), *Candida albicans* strain 74 (*C. albicans*) and *Pseudomonas aeruginosa* strain 1390 (*P. aeruginosa*) were supplied by the National Bank for Industrial Microorganisms and Cell Cultures (NBIMCCs), Bulgaria.

### 2.2. Preparation and Characterization of Electrospun Composite Fibrous Materials from PEO/PLA/BW

Composite fibrous materials from PEO/PLA/BW were prepared by electrospinning at the following weight ratios between the partners: PEO(80)/PLA(10)/BW(10), PEO(70)/PLA(15)/BW(15), PEO(60)/PLA(20)/BW(20) and PEO(50)/PLA(25)/BW(25). The components were dissolved in chloroform at a constant PEO concentration of 2.8% (*w*/*v*). For example, a spinning solution of PEO(70)/PLA(15)/BW(15) was prepared by dissolving 0.15 g PLA in 25 mL chloroform using a magnetic stirrer. After the complete dissolution of PLA, 0.15 g BW was added under vigorous stirring, followed by the addition and dissolution of 0.7 g PEO. For the electrospinning experiments, the feed rate for delivering the solution was 3 mL/h. The formed fibers were collected on a rotating drum collector (collector rotation speed—2200 rpm), and the tip-to-collector distance was 20 cm. The applied voltage for the electrospinning was 10 kV, provided by a high voltage generator for scientific use, Model: HVG-CONT-LCD, Linari Engineering (Pisa, Italy); the experiments were performed at room temperature (22 ºC), and at a relative humidity of 53%. For comparison, PEO(75)/PLA(25) and PEO(70)/BW(30) mats were prepared by electrospinning as well, using the same electrospinning conditions.

The dynamic viscosity of PEO/PLA/BW solutions was determined at 25 ± 0.1 ºC by a cone/plate Bookfield DV-II+ programmable viscometer (Middleboro, MA, USA).

The morphology of the fibers in the electrospun mats was assessed by scanning electron microscopy (SEM). The fibrous samples were vacuum-coated with gold and analyzed by a Philips 515 SEM (Tokyo, Japan). The criteria for the complex evaluation of electrospun mats [39] were applied for assessment of the fibers’ morphology and determination of the mean fiber diameter. Image J software was employed and at least 30 fibers from SEM images were used for estimation of the mean fiber diameters. Transmission electron microscopy (TEM) was employed for assessing the core/double-sheath structure of the fibers (individual fibers were deposited on a copper grid) using a JEM 2100 TEM (JEOL Co., Ltd., Tokyo, Japan), operating voltage 200 kV.

Attenuated total reflection Fourier transform infrared (ATR-FTIR) spectroscopic analyses were performed using a Nicolet™ iS™50 spectrometer (Thermo Fisher Scientific Inc., Waltham, MA, USA) equipped with an IR diamond iS50 ATR (diamond crystal, depth of penetration of the IR beam into the sample ca. 2 µm) accessory. The spectra were recorded from 4000 cm^−1^ to 400 cm^−1^ with a spectral resolution of 2 cm^−1^ using OMNIC software, and the spectra were corrected for H_2_O and CO_2_.

Differential scanning calorimetry (DSC) using a Discovery DSC 250 (TA, Instruments, New Castle, DE, USA) or Perkin Elmer DSC 8500 was performed for assessment of the thermal behavior of the fibrous materials: heating from 0 to 200 °C, with a heating rate of 10 °C/min under nitrogen flow. The first heating run was used for estimation of the melting temperatures (T_m_) and the fusion enthalpies (ΔH_m_).

The amorphous/crystalline structure of PEO/PLA/BW fibrous materials was evaluated by X-ray diffraction (XRD) analyses (computer-controlled D8 Bruker Advance diffractometer) at 2*θ* range from 5º to 80º with a step of 0.02º, and a counting time of 1 s/step.

The static water contact angle of the fibrous materials was determined by an Easy Drop DSA20E Krűss GmbH apparatus (Hamburg, Germany; volume of the water drop—6.25 μL). The water contact angle values were estimated using the Easy Drop DSA20E software measuring the contact angle of 10 different water drops deposited on each sample.

X-ray photoelectron spectroscopy (XPS) was applied for evaluation of the chemical composition of the mats’ surfaces using an ESCALAB-MkII (VG Scientific) spectrometer with a monochromatic Mg Kα X-ray source. The energy calibration was performed using C1s line at 285 eV.

The mechanical properties of PEO/PLA/BW mats were assessed by performing tensile tests (INSTRON 3344 having a 50 N load cell, crosshead speed—20 mm/min) at room temperature and a relative humidity of 53%. Rectangular specimens (20 mm × 60 mm) of the PEO/PLA/BW mats were cut in the collector rotation direction, and the thickness of the specimens was determined by a Digital Thickness Gauge FD 50 (Käfer GmbH, Bremen, Germany). The mean values of the Young’s modulus, tensile strength and elongation at break were determined based on the regression of the linear part of the stress-strain curves from at least 10 tested specimens of each mat.

The use of purposely designed cells, described in detail in [37], for the fixation of the fibrous materials enabled the evaluation of the weight loss of the mats in an aqueous medium. Immersion of a mat (4.5 mg) in 30 mL hexane for 24 h was performed in order to extract BW from PEO/BW/NQ fibrous materials.

### 2.3. Preparation and Characterization of Electrospun Fibrous Materials from PEO/PLA/BW/NQ

Composite fibrous materials made from PEO/PLA/BW/NQ were prepared by electrospinning at the following weight ratios: PEO(80)/PLA(10)/BW(10), PEO(70)/PLA(15)/BW(15), PEO(60)/PLA(20)/BW(20) or PEO(50)/PLA(25)/BW(25) (NQ content—10 wt.% with respect to the total weight of the solids; solvent—chloroform; PEO concentration—2.8% (*w*/*v*)). For example, a spinning solution of PEO(70)/PLA(15)/BW(15)/NQ was prepared by dissolution of 0.15 g PLA in 25 mL chloroform, followed by addition of 0.11 g NQ and 0.15 g BW. After the complete dissolution of BW, 0.7 g PEO was added under vigorous stirring. The obtained solution was electrospun under the above-described conditions (feed rate, applied field strength, temperature, humidity) for the preparation of PEO/PLA/BW mats.

The obtained PEO/PLA/BW/NQ fibrous materials were characterized by SEM, TEM, XRD, determination of the water contact angle, XPS and tensile tests using the methods described in detail above.

The actual NQ loading content was determined by UV–vis spectrophotometry. Samples of PEO/PLA/BW/NQ mats were dissolved in chloroform, and the amount of loaded NQ was estimated at a wavelength of 350 nm by a Beckman Coulter DU 800 UV–Vis spectrophotometer. The loading efficiency (%) was estimated using the following equation: Loading efficiency (%) = 100 × actual loading/theoretical loading. The experiments were performed in triplicate.

The release of NQ was performed in vitro in a phosphate buffer (NaH_2_PO_4_/KH_2_PO_4_) with pH 7.4 (ionic strength 0.1) at 37 ºC. Mats (3 mg; 10 × 10 mm) were immersed in 100 mL buffer solution and the samples were thermostated in a shaker bath (Julabo, Seelbach, Germany) at 37 ºC using the cell [37] for fixation of the fibrous materials. The release of NQ was evaluated by withdrawing aliquots from the solution at certain time intervals and recording their absorbance by a DU 800 UV–vis spectrophotometer (Beckman Coulter) at a wavelength of 447 nm. The amount of the released NQ over time was calculated using calibration curves in phosphate buffer (NaH_2_PO_4_/KH_2_PO_4_, pH 7.4, ionic strength 0.1) with correlation coefficient R~0.999. The data were average values from three measurements.

### 2.4. Assessment of the Behavior of Electrospun Composite Materials from PEO/PLA/BW and PEO/PLA/BW/NQ in Contact with Pathogenic Microorganisms

The antibacterial and antifungal activities of PEO/PLA/BW/NQ mats were evaluated against Gram-positive (*S. aureus*) and Gram-negative (*E. coli* and *P. aeruginosa*) bacteria and against the fungus *C. albicans*. PEO(50)/PLA(25)/BW(25) was used as a control mat. All electrospun samples were prepared in the form of discs (diameter 10 mm) with an NQ content of ca. 200 µg. A solid agar nutrient medium containing 4% glucose, 2% pectin, 0.1% yeast extract and 1.2% Sabouraud agar was used in the studies. The medium surface was inoculated with a suspension of a 24 h cell culture of *S. aureus*, *E. coli*, *P. aeruginosa* or *C. albicans* (5 × 10^5^ cells/mL). After inoculation (5–10 min), the discs were placed on the inoculated surface (two discs of each sample in a Petri dish). The Petri dishes were incubated at 37 ºC for 24 h for *S. aureus*, *E. coli* and *P. aeruginosa*, and for 48 h for *C. albicans*, after which the zones of inhibition around each disc were measured. The average values of the zones of inhibition were determined using the Image J software, based on ten measurements.

### 2.5. Statistical Analysis

All experiments were performed in triplicate, and the results are presented as means and standard deviations (±SD). A statistically significant difference was found by one-way analysis of variance (ANOVA), followed by Bonferroni’s test using GraphPAD PRISM software, Version 5 (GraphPad Software Inc., San Diego, CA, USA).

## 3. Results and Discussion

### 3.1. Composite PEO/PLA/BW Fibrous Materials Prepared by Electrospinning at Different Weight Ratios between the Partners

Using chloroform as a common solvent for PEO, PLA and BW allowed us to obtain homogeneous blend solutions of PEO, PLA and BW that we subjected to single-spinneret electrospinning. Fibrous materials of PEO/PLA/BW were prepared at the following weight ratios: PEO(80)/PLA(10)/BW(10), PEO(70)/PLA(15)/BW(15), PEO(60)/PLA(20)/BW(20) and PEO(50)/PLA(25)/BW(25).

**Figure 1:**
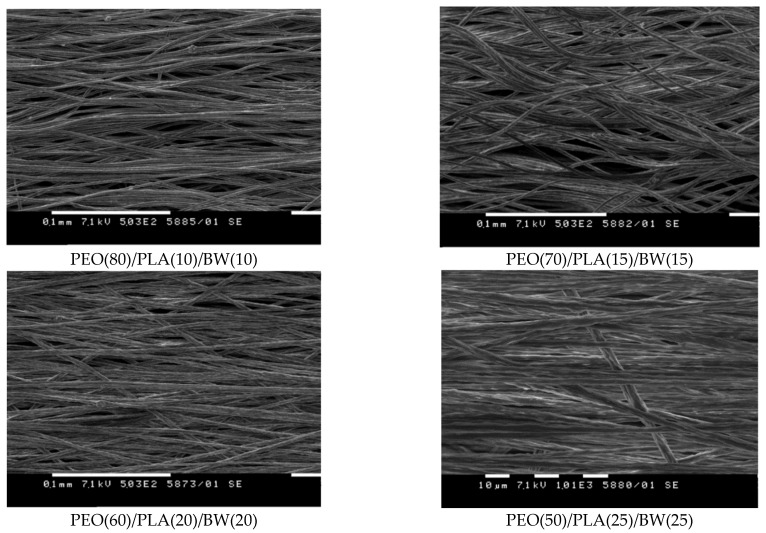
SEM micrographs of PEO/PLA/BW fibrous materials prepared by electrospinning at different weight ratios (shown under each micrograph) between the partners and at equal PEO concentration in the spinning solution. Magnification: ×500.

SEM micrographs of fibrous materials are presented in Figure 1. As seen, the fibers were cylindrical in shape, and no defects were present along their length. The dependence of the mean fiber diameter of the PEO/PLA/BW system on the content of PLA in the spinning solution is shown in the Supplementary Material, Figure S1. As seen, the mean fiber diameter showed a tendency to increase in value with increasing PLA content, from 2.2 ± 0.35 µm for PEO fibers to 3.3 ± 1.2 µm for PEO(50)/PLA(25)/BW(25) fibers. In addition, the increase in PLA content resulted in fibers with a broader diameter distribution (Supplementary Material, Figure S1).

This was attributed to the increase in the solution viscosity in the presence of PLA (BW, as a material composed of low-molecular-weight substances, did not affect the viscosity value of the spinning solutions). As seen from the inset dependence in Supplementary Material, Figure S1, the solutions that contain PEO/PLA/BW had higher values of dynamic viscosity as compared to the PEO solution, and the highest value of the dynamic viscosity (400 ± 10 cP) was registered by the PEO(50)/PLA(25)/BW(25) solution. Due to this solution having the highest viscosity, its electrospinning led to obtaining fibers with the highest mean diameter value and the broadest diameter distribution (Supplementary Material, Figure S1).

The water contact angle of the new fibrous materials from the PEO/PLA/BW series was determined, and it was found that PEO(80)/PLA(10)/BW(10) materials were hydrophobic with a water contact angle of 101 ± 8.85º. A further increase in PLA and BW content did not led to a significant increase in the water contact angle value (ca. 118 ± 7.60º). In conclusion, the presence of the hydrophobic PLA and BW in the solutions subjected to electrospinning resulted in imparting of hydrophobicity to the surface of PEO/PLA/BW mats.

**Figure 2:**
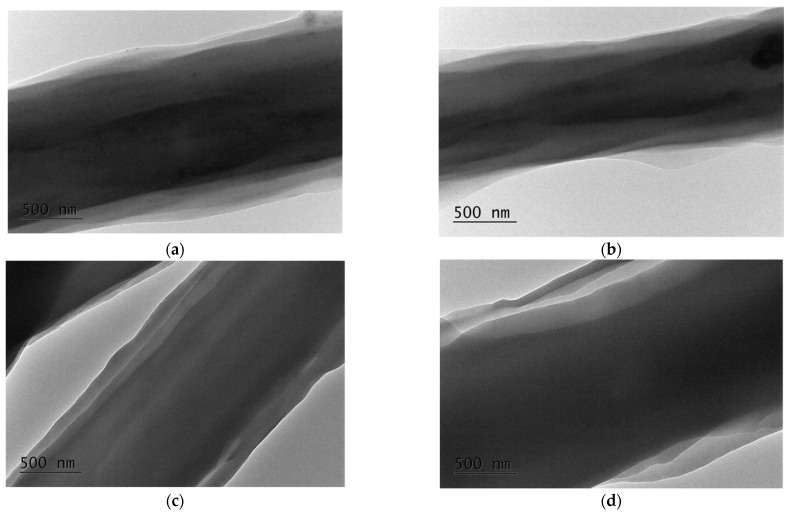
TEM micrographs of a PEO/PLA/BW fiber prepared at weight ratios of PEO(80)/PLA(10)/BW(10) (**a**), PEO(75)/PLA(15)/BW(15) (**b**), PEO(60)/PLA(20)/BW(20) (**c**) and PEO(50)/PLA(25)/BW(25) (**d**). Magnification: ×10,000.

Previously, we showed that the electrospinning of PEO/BW led to obtaining composite core-sheath-like fibers composed of a PEO core and a BW sheath [37]. Taking into account that PEO is a hydrophilic polymer, and that both PLA and BW are hydrophobic, we had expected that the PEO/PLA/BW system would result in fibers having a core-sheath-like structure, as well. TEM micrographs of fibers prepared by electrospinning of PEO(80)/PLA(10)/BW(10), PEO(70)/PLA(15)/BW(15), PEO(60)/PLA(20)/BW(20) and PEO(50)/PLA(25)/BW(25) solutions are given in Figure 2. Noteworthy is the fact that a single core and two sheaths are observed. The preparation of core-sheath fibers from a homogeneous solution of polymer/low-molecular-weight substance or from two polymers has been reported, and this is attributed to phase separation driven by differences in the molecular weights of both partners [14,15,16,17]. Having this in mind, as well the molar masses of the partners in the present study, we made the assumption that the core was composed of PEO, the inner sheath of PLA and the outer sheath of BW. The areas of the core and both sheath segments, as well as the sheath-to-core area ratio, were calculated from the recorded TEM micrographs (Table 1).

As seen, the increase in PLA and BW content in the spinning solution led to fibers in which the sheath-to-core area ratio increased from 0.24 to 0.63, for fibers made of PEO(80)/PLA(10)/BW(10) and PEO(50)/PLA(25)/BW(25), respectively. This was an indication that the higher the PLA and BW content in the solution, the thicker the sheath of the resulting fibers.

**Figure 3:**
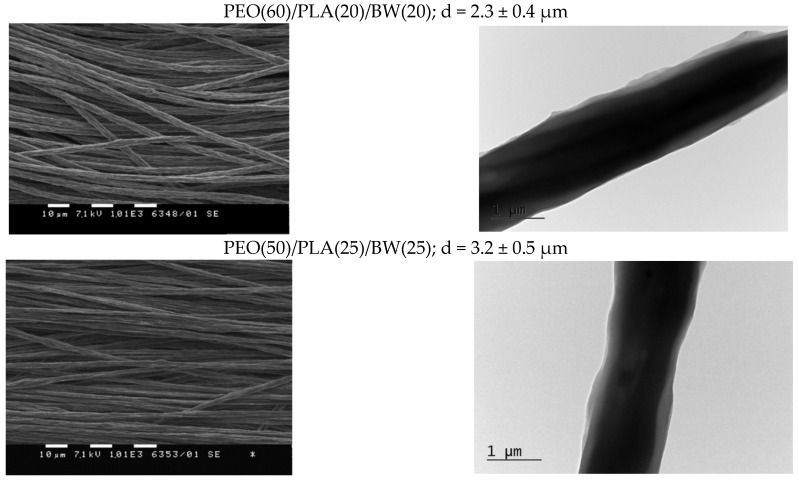
SEM micrographs (magnification: ×1000) of mats and TEM micrographs (magnification: ×5000) of a single fiber of PEO(60)/PLA(20)/BW(20) and PEO(50)/PLA(25)/BW(25) after their 24 h stay in hexane.

In order to reveal the architecture of the PEO/PLA/BW fibers, selective extraction of BW in hexane and of PEO in water was performed. Since BW is soluble in hexane, while PEO and PLA are not soluble, we assumed that that the immersion of PEO/PLA/BW mats in hexane would lead to dissolution of the outer BW sheath, resulting in core-sheath fibers consisting of a PEO core and PLA sheath. The weight loss of the mats after a 24 h immersion in hexane was determined, and was compared with the theoretical weight loss. The latter was calculated based on the assumption that all of the BW had been extracted from the mats in hexane. As seen from Supplementary Material, Figure S2, the experimentally determined weight loss was close to the theoretical one (i.e., the 24 h stay of the mats in hexane resulted in almost complete extraction of BW from the fibrous materials). The performed SEM analyses revealed that the mean diameter of the fibers from PEO(60)/PLA(20)/BW(20) and PEO(50)/PLA(25)/BW(25) mats after their 24 h stay in hexane was not significantly altered (the mean diameter values are given in Figure 3). The TEM micrographs of a single fiber showed that the fibers were composed of a core and a single sheath after the extraction in hexane (Figure 3). Using the TEM micrographs shown in Figure 3, the sheath-to-core area ratio was estimated. It was found that it was less than that calculated for the pristine fibers (before treatment with hexane). For the PEO(60)/PLA(20)/BW(20) sample, the sheath-to-core area ratio was 0.20, while it was 0.49 before treatment with hexane (Table 1). For PEO(50)/PLA(25)/BW(25) mats, the sheath-to-core area ratio was 0.40, while it was 0.63 before treatment with hexane (Table 1). The observation of a single sheath by TEM, as well as the decrease in the sheath-to-core area ratio, were indications that the outer sheath consisted of BW. Additional evidence that the fibers were composed of a PEO core and PLA sheath were found by determining the water contact angle of mats treated with hexane. The treated mats remained hydrophobic, having a water contact angle of ca. 109 ± 7º. This was a significant difference compared to mats from the PEO/BW system. For the latter, the extraction of BW from the mats led to its removal, and to observation of immediate dissolution of the fibrous materials composed only of PEO in contact with aqueous medium [37]. The obtained result evidenced the presence of a PLA sheath in the PEO/PLA/BW fiber’s structure. At the next stage, after extraction with hexane, PEO(60)/PLA(20)/BW(20) mats were immersed for 48 h in distilled water in order to find whether they would preserve their fibrous structure after the removal of PEO in the aqueous medium. This would be an indication that after extraction with hexane, the fibers consisted of a PEO core and PLA sheath. It was found that due to the swelling and the removal of PEO from the fibers, the mats’ stay in distilled water led to sticking of the fibers (Supplementary Material, Figure S3a). As seen from the TEM micrographs shown in Supplementary Material, Figure S3b,c, two types of fibers could be distinguished: one of them had core-sheath structure, while the pale gray ones consisted only of the PLA sheath of the fibers.

The FTIR spectra of BW pellets, PLA mats, PEO mats and PEO(60)/PLA(20)/BW(20) mats before and after treatment with hexane alone (24 h), or after subsequent treatment with water (48 h), are shown in Supplementary Material, Figure S4. For BW, the following observations were made: highly pronounced characteristic bands at 2915 and 2848 cm^−1^ (stretch vibrations of CH_3_ and CH_2_ groups from the hydrocarbon chains of the compounds involved in BW composition); a broad characteristic band at 1735 cm^−1^ (a stretch vibration of the BW ester C=O group); a shoulder at 1723 cm^−1^ (a stretch vibration of the acid C=O group from the fatty acids in BW composition); and a characteristic band at 1170 cm^−1^ (a stretch vibration of the C-O-C group from the esters in BW composition). For PLA mats, the following were detected: a weakly intensive characteristic band at 2997 and 2944 cm^−1^ (stretch vibrations of PLA CH_3_ and CH groups); an intensive characteristic band at 1748 cm^−1^ (a stretch vibration of the PLA ester C=O group); and a characteristic band at 1080 cm^−1^ (a stretch vibration of the PLA ester C-O-C group). For PEO mats, the following were registered: a broad characteristic band at ca. 2880 cm^−1^ (a stretch vibration of PEO CH_2_ groups), as well as a highly pronounced characteristic band at 1096 cm^−1^ (a stretch vibration of PEO C-O-C groups). Bands characteristic of both PEO and BW were detected in the FTIR spectrum of PEO(60)/PLA(20)/BW(20) mats, in the range 3000–2800 cm^−1^. With respect to the C=O group in the spectrum of PEO(60)/PLA(20)/BW(20) mats, a characteristic band at 1750 cm^−1^ (stretch vibrations of the PLA ester C=O group and BW ester C=O group), as well as a shoulder at 1735 cm^−1^ (a stretch vibration of the acid C=O group from the fatty acids in BW composition) were detected. The extraction with hexane led to the removal of BW from PEO(60)/PLA(20)/BW(20) mats, as evidenced by the lack of characteristic bands for BW CH_3_ and CH_2_ groups in the range 3000–2800 cm^−1^, as well from the absence of a shoulder at 1723 cm^−1^ (a stretch vibration of the acid C=O group from the fatty acids in BW composition). After the extraction with hexane, the presence of PEO in PEO(60)/PLA(20)/BW(20) mats was evidenced by the detection of a broad band in the range 3000–2800 cm^−1^, characteristic for PEO CH_2_ groups. The subsequent extraction with hexane and water led to removal of PEO as evidence by the lack of a broad band in the range 3000–2800 cm^−1^, characteristic for PEO CH_2_ groups.

**Figure 4:**
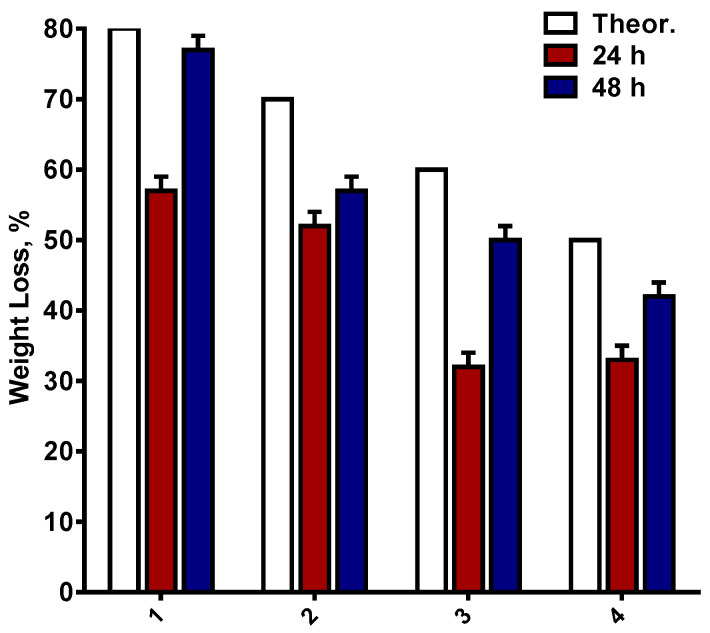
Dependence of the weight loss of PEO(80)/PLA(10)/BW(10) (1), PEO(70)/PLA(15)/BW(15) (2), PEO(60)/PLA(20)/BW(20) (3) and PEO(50)/PLA(25)/BW(25) (4) mats after a 24 or 48 h stay in distilled water. For comparison, the theoretical value of the weight loss, calculated based on the assumption that all PEO has been extracted from the corresponding mat in distilled water, is given as well.

With respect to the selective extraction of PEO in water, the weight loss of PEO/PLA/BW fibrous materials after immersion in distilled water depending on the time of immersion (24 and 48 h) was determined (Figure 4).

For comparison, the theoretical value of the weight loss, calculated based on the assumption that all PEO has been extracted in distilled water, is given as well. As seen from Figure 4, the weight loss of the mats after a 24 h stay in distilled water was lower than the theoretical value. This is an indication that this period of time is not enough for extracting all of the PEO from the fibers. For comparison, for PEO/BW mats, it was found gravimetrically that all of the PEO had been removed from the fibrous materials after a 24 h stay in aqueous medium [37]. This indicated that the presence of an inner PLA sheath in the PEO/PLA/BW fibers led to a delay in the extraction of PEO from the fiber’s core. The experimentally determined weight loss of PEO/PLA/BW mats approximates the theoretical value after a 48 h stay of the fibrous materials in water. Therefore, almost all of the PEO had been extracted in the aqueous medium after this period of time. In order to evaluate the effect of the stay in distilled water on the fiber morphology, freeze-dried PEO(80)/PLA(10)/BW(10) and PEO(50)/PLA(25)/BW(25) mats were observed by SEM (Supplementary Material, Figure S5). As seen, for the PEO(80)/PLA(10)/BW(10) mat (for which the PLA and BW content was the lowest) the 24 h stay in distilled water led to swelling and sticking of the fibers; however, their fibrous structure had been preserved. After a 48 h stay in water, a loss of the fibrous structure of the mat is observed. For the mats with the highest PLA and BW content (i.e., PEO(50)/PLA(25)/BW(25)) the 24 h stay in water led to a preservation of their fibrous structure, and to insignificant alteration of the fibers’ morphology and mean diameter values. Again, preservation of its fibrous structure is observed after a 48 h stay in water, and the fibers are in a swollen state. Determination of the mean fiber diameter was hindered because of the presence of fibers stuck together. In conclusion, PEO/PLA/BW mats possess improved behavior after staying in an aqueous medium, as compared to PEO/BW mats.

**Figure 5:**
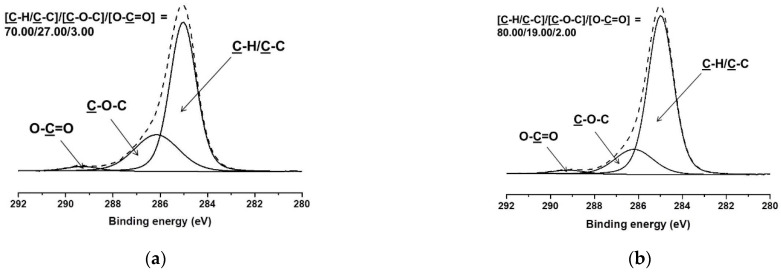
C1s spectra of PEO(70)/PLA(15)/BW(15) (**a**) and PEO(50)/PLA(25)/BW(25) (**b**) mats.

In order to further demonstrate that the outer fiber sheath is composed of BW, we evaluated the surface chemical composition of the PEO/PLA/BW fibrous materials by XPS. Carbon and oxygen were detected in the survey XPS spectra. The estimated C/O ratio for a PEO mat was 67/33, for a PLA mat was 60/40 and for BW pellets was 97/3. It was found that for PEO(70)/PLA(15)/BW(15) mats, the experimentally determined C/O ratio was 92/8, and that it was higher than the theoretically calculated C/O ratio of 70/30. This can be attributed to the presence of BW on the surface of the fibers. Further increases in the PLA and BW content of the mats led to additional increases in the C/O ratio, up to 95/5 for PEO(50)/PLA(25)/BW(25) fibrous materials (for these mats the theoretical C/O ratio is 72/28). From the results obtained by the survey XPS spectra, it can be concluded that the outer sheath of the core/double-sheath fibers was composed of BW. Additional evidence was found in the C1s spectra of PEO(70)/PLA(15)/BW(15) and PEO(50)/PLA(25)/BW(25) mats (Figure 5).

Theoretically, for PEO(70)/PLA(15)/BW(15) fibrous material, the [C-H/C-C]/[C-O-C]/[O-C=O] ratio is 19.00/75.00/6.00. As seen from Figure 5, the experimentally estimated ratio showed that the content of carbon atoms engaged in the C-H/C-C group on the surface of the fibers reached up to 70 and 80%, for PEO(70)/PLA(15)/BW(15) and PEO(50)/PLA(25)/BW(25) mats, respectively. Such a type of atom originates from the long aliphatic chains that are present in the constituents of BW. The increasing BW content of PEO(50)/PLA(25)/BW(25) mats resulted in an increased proportion of carbon atoms engaged in the C-H/C-C group, and again the experimentally estimated [C-H/C-C]/[C-O-C]/[O-C=O] ratio (Figure 5B) was higher than the theoretical value (32.00/59.00/9.00). The obtained XPS results revealed that the surface chemical composition of PEO/PLA/BW fibrous materials approximated BW composition. This is additional evidence that the outer sheath of the fibers consists of BW.

The obtaining of PEO/PLA/BW fibers with core/double-sheath structure can be attributed to the occurrence of spontaneous phase separation of the partners during the electrospinning, driven by the significant differences in the molar masses of PEO, PLA and BW, the incompatibility of the partners, as well as by the air hydrophobicity. PEO has the highest molar mass (M_η_ = 600,000 g/mol), PLA has lower molar mass (M_w_ = 259,000 g/mol) and BW is composed of low-molecular-weight substances. We assumed that under the action of the electric field during the electrospinning process, BW as a complex mixture of low-molecular-weight substances would most easily migrate to the surface of the spinning jet and, accordingly, to the surface of the resulting fibers. The presence of an inner shell of PLA was most likely due to the lower molar mass of this polymer compared to PEO. Therefore, PLA more easily migrated to the surface of the polymer jet, but had more difficultly compared to BW. Most probably, BW assisted in obtaining the inner PLA sheath of the fibers. The hypothesis that a small fraction of PLA could be located in the PEO core, as well as a small fraction of PEO in the PLA inner sheath, should not be excluded. With respect to BW, the presence of traces of it in the core and inner sheath is also possible.

**Figure 6:**
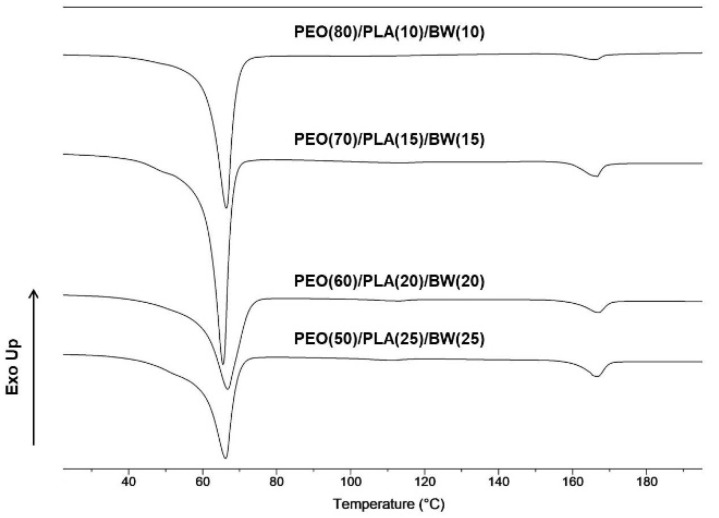
DSC thermograms (first heating run) of PEO/PLA/BW fibrous materials prepared at different weight ratios, registered using Discovery DSC 250 (TA, Instruments, New Castle, DE, USA).

The thermal characteristics of PEO/PLA/BW fibrous materials were evaluated by DSC. DSC thermograms of the mats are presented in Figure 6.

Two melting peaks at ca. 65 ºC and 166 ºC were registered in DSC thermograms. The first peak is attributed to the melting of PEO and BW. PEO and BW melt in a very close temperature range: PEO is characterized by a narrow melting peak and has melting temperature of ca. 65 ºC, while BW melts in a broad temperature range (from ca. 40 ºC to 70 ºC). This makes it difficult to distinguish the melting peaks of PEO and BW. The registered second peak at 166 ºC in the thermograms was attributed to PLA melting in the composite PEO/PLA/BW fibrous materials. As seen from Figure 6, upon increasing the BW content in the composite fibrous materials, the melting peak at ca. 65 ºC broadened. The melting enthalpies of PLA and PEO/BW in the composite fibrous materials as determined by DSC are listed in Table 2. Using the PLA melting enthalpy, the PLA crystallinity in the composite fibers was estimated, taking into account the PLA weight fraction in the mat. As seen, for PEO/PLA/BW fibrous materials, the PLA crystallinity degree was close to 50%.

**Figure 7:**
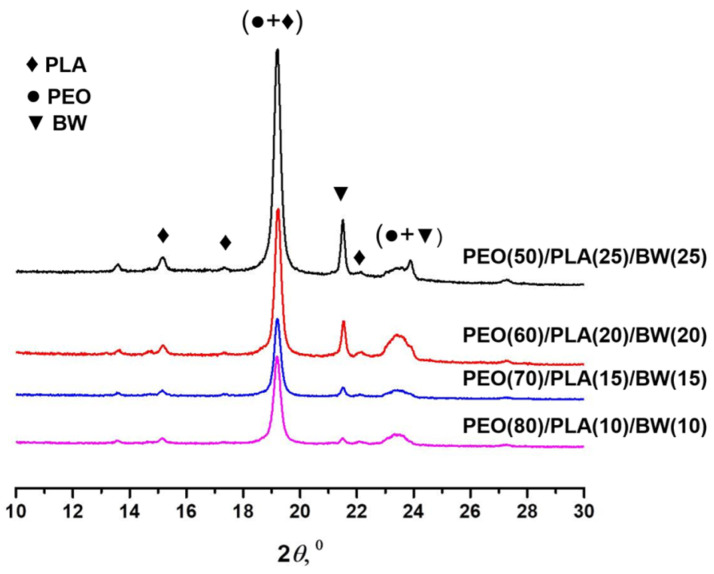
XRD patterns of materials prepared at different ratios of PEO/PLA/BW.

The amorphous/crystalline structure of PEO/PLA/BW fibrous materials was evaluated by X-ray diffraction analyses (XRD). The registered XRD patterns are shown in Figure 7. PEO is a semi-crystalline polymer, having diffractions at 19.2º and 23.3º [41]. Diffractions at 21º and 24º are present in XRD patterns of BW [42,43].

PLA is semi-crystalline aliphatic polyester, and diffractions at 14.8º, 16.7º, 19.1º and 22.3º are present in its XRD patterns [44]. As seen from Figure 7, the intensity of the diffraction at 21.5º, characteristic for BW, increased with increasing BW weight fraction in the fibrous materials. This result was in accordance with the previously found dependence for PEO/BW fibrous materials [37]. The overlapping of PEO and PLA diffractions at ca. 19º did not allow us to distinguish the presence of crystalline phases of PEO and PLA at this 2*θ* value. The presence of the crystalline phase of PLA was evidenced by the recorded DSC thermograms, as well as by the presence of other diffraction characteristics for PLA in XRD patterns (in the range of 2*θ* from 14 to 18º). From the obtained results, it can be concluded that there was a crystalline phase for each of the components (PEO, PLA and BW) in the PEO/PLA/BW fibrous materials. Additional evidence for the removal of BW from the PEO/PLA/BW fibrous materials after extraction with hexane was found in the XRD patterns of the fibrous materials after their 24 h stay in hexane (Supplementary Material, Figure S6). Diffraction at 21º, characteristic of BW, was lacking in the XRD patterns; in other words, most of the BW was extracted from the mats.

**Figure 8:**
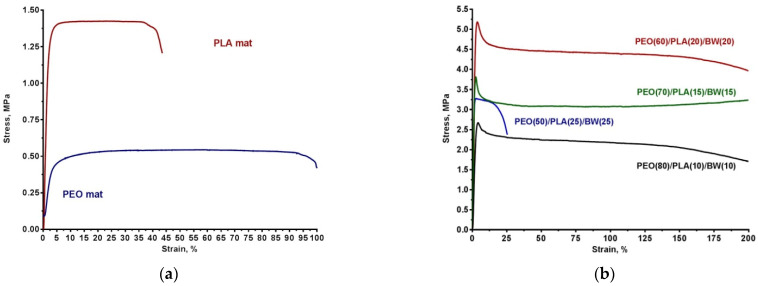
Stress-strain curves for electrospun materials from PEO or PLA (**a**), and for the PEO/PLA/BW series (**b**).

**Figure 9:**
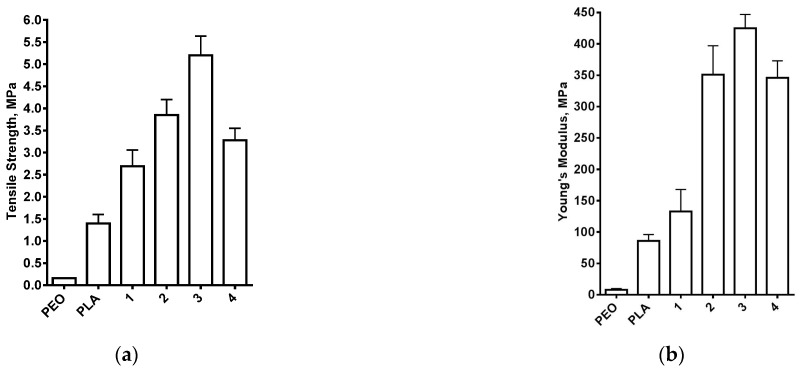
Tensile strength (**a**) and Young’s modulus (**b**) of PEO(80)/PLA(10)/BW(10) (1); PEO(70)/PLA(15)/BW(15) (2); PEO(60)/PLA(20)/BW(20) (3) and PEO(50)/PLA(25)/BW(25) (4) mats. For comparison, the values of these parameters are given for PEO and PLA mats, as well.

The stress-strain curves of the control mats from PEO and PLA are presented in Figure 8a. The PEO mat was a flexible material having tensile strength (σ) 0.55 ± 0.06 MPa, Young’s modulus (E) 17± 2 MPa and elongation (ε) ca. 90%. PLA electrospun material had higher values of tensile strength (1.40 ± 0.20 MPa) and Young’s modulus. However, this material was brittle, having an elongation of ca. 35%. Noteworthy was the fact that a break of the specimens from both PEO and PLA mats was detected during the tensile tests, after reaching the maximum elongation. For PEO/PLA/BW mats prepared at a weight ratio of 80/10/10, 70/15/15 or 60/20/20 during the tensile tests, necking was observed, and the specimens underwent significant elongation (Figure 8b) and did not break even after elongation of 200% (a digital image of a highly elongated specimen is shown in Supplementary Material, Figure S7b). It has been reported that for PLA/PEO films, the presence of the flexible polyether contributes to the increase in the elongation of the composite polymer materials [45]. A similar increase in the elongation of PEO-containing polymer materials has also been reported for electrospun materials from a poly(3-hydroxybutyrate-*co*-3-hydroxyvalerate)/PEO pair [46], and the elongation increases as the PEO content increases. PEO/PLA/BW mats prepared at a weight ratio of 80/10/10, 70/15/15 or 60/20/20 had significantly higher σ and E values as compared to both PEO and PLA mats (Figure 9).

The higher σ and E values of PEO/PLA/BW mats, as compared to PLA mats, can be attributed to the differences in the amorphous/crystalline phase of the aliphatic polyester in PLA fibrous material and in PEO/PLA/BW mats. It is known that the electrospinning of PLA leads to the preparation of a mat in which the polymer is in the amorphous state [40,47]. This was also found in the present study: only an amorphous halo was detected in the XRD patterns of PLA mat. For PEO/PLA/BW mats, ca. 50% of incorporated PLA is in the crystalline phase, as evidenced by the DSC results (Figure 6, Table 2). We attribute the higher σ and E values detected for PEO/PLA/BW mats, as compared to PLA fibrous material, to the presence of a crystalline phase of PLA. As seen in Figure 9, for PEO/PLA/BW mats prepared at weight ratios from 80/10/10 to 60/20/20, σ and E values increase with increasing PLA and BW content, reaching values close to 5.5 MPa and 425 MPa, respectively, for PEO(60)/PLA(20)/BW(20) mats. This can be attributed to the increase in PLA content, as this is the component that has a positive effect on the value of these two parameters. The high flexibility of these fibrous materials is attributed to the presence of PEO in the mats. For PEO(50)/PLA(25)/BW(25) fibrous specimens, σ and E values are lower compared to PEO(60)/PLA(20)/BW(20) mats, and the former undergo insufficient elongation: 20 ± 1% (Figure 8b) followed by breaking of the specimens (Supplementary Material, Figure S7c). However, again, the σ and E values of PEO(50)/PLA(25)/BW(25) mats are significantly higher than those of PEO and PLA mats. It can be concluded that the presence of core/double-sheath fibers in the mats imparts to the latter improved mechanical behavior compared to PEO and PLA mats. The lower σ and E values of PEO(50)/PLA(25)/BW(25) mats and their higher brittleness compared to the mats prepared at the other ratios can probably be attributed to PEO(50)/PLA(25)/BW(25) having the highest weight fraction of the brittle BW. In order to determine the contribution of PLA to the improved mechanical behavior of fibrous materials composed of PLA/PEO/BW, we compared the mechanical parameters of PEO(70)/BW(30) mats with fibrous materials in which 15% of the BW content had been replaced by PLA (i.e., PEO(70)/PLA(15)/BW(15) mats). It was found that PEO(70)/BW(30) mats had lower mechanical parameters compared to PEO(70)/PLA(15)/BW(15) mats: σ of 0.40 ± 0.04 MPa (σ value is close to 4 MPa for PEO(70)/PLA(15)/BW(15) mats), E of 18 ± 2 MPa (E value is close to 350 MPa for PEO(70)/PLA(15)/BW(15) mats) and ε of ca. 53% (ε value is higher than 200% for PEO(70)/PLA(15)/BW(15) mat); moreover, a break of the PEO(70)/BW(30) specimens was detected after reaching maximum elongation. Therefore, the presence of PLA in the mats is the reason for the significant increase in the mechanical behavior of the fibrous materials based on PEO and BW. Taking into account of the fact that PEO(60)/PLA(20)/BW(20) exhibited the best mechanical performance, it can be concluded that this composition of the fibrous materials was the optimum ratio.

### 3.2. Composite PEO/PLA/BW/NQ Fibrous Materials Prepared by Electrospinning

In order to demonstrate that the new fibrous materials can be used as drug carriers, 5-nitro-8-hydroxyquinoline (NQ) was selected as a model drug, as it has highly pronounced antibacterial and antifungal activity. PEO(80)/PLA(10)/BW(10)/NQ, PEO(70)/PLA(15)/BW(15)/NQ, PEO(60)/PLA(20)/BW(20)/NQ and PEO(50)/PLA(25)/BW(25)/NQ mats were prepared by electrospinning (NQ content was 10% of the weight respect to the total weight of the solids). Owing to the good solubility of NQ in chloroform, it was incorporated into the spinning solution that contained PEO, PLA and BW. The actual loading of NQ (98 ± 1.0%) was very close to the theoretical value. Representative SEM micrographs of PEO/PLA/BW/NQ fibrous materials are shown in Supplementary Material, Figure S8. For PEO/PLA/BW/NQ fibrous materials, it was found that they consisted of main fibers, and whiskers arising from the main fibers. The mean diameter of the main fibers (their values are given in Supplementary Material, Figure S8) did not differ significantly from the diameter of the fibers that do not contain NQ. The mean diameter value of the observed whiskers did not depend on PEO/PLA/BW ratio and was ca. 600 nm. Similar to the PEO/BW/NQ system [37], the phenomenon of whisker formation was attributed to the simultaneous presence of BW and NQ in the spinning solution, and was attributed to ionic imbalance on the surface of the spinning jet leading to ejection of thinner jets from the main jet, and subsequent whisker formation. The TEM analyses (Supplementary Material, Figure S9) showed that the fibers from the PEO/PLA/BW/NQ series are composed of a core and a double sheath. Therefore, the presence of NQ did not lead to disruption of the formation of core/double-sheath structure during the electrospinning process. The XPS analyses showed that for PEO/PLA/BW/NQ mats, the surface was enriched in carbon atoms engaged in C-C/C-H bonds. This indicated that there was BW on the surface of the fibers in this case, as well. Only traces of NQ were found on the fiber’s surface, and the amount did not exceed 1%, as evidenced by XPS analysis. PEO/PLA/BW/NQ fibrous materials were hydrophobic (Supplementary Material, Figure S10), and the recorded water contact angle was 115 ± 5.86°. Hence, the incorporation of NQ in the mats did not alter the hydrophilic/hydrophobic behavior of the fibrous materials from the PEO/PLA/BW series.

The XRD patterns of PEO/PLA/BW/NQ fibrous materials prepared at different weight ratios between the polymers and BW are presented in Supplementary Material, Figure S11a. With respect to NQ, the most intensive diffractions of the drug (Supplementary Material, Figure S11b) were at 2*θ* values in the range from 10 to 15 º, and it was difficult for them to be distinguished in the XRD patterns of PEO/PLA/BW/NQ mats, since they fell within the detected amorphous halo of PLA. The DSC analyses (Supplementary Material, Figure S12) revealed that the crystallinity degree of PLA in the mats is ca. 35%, i.e., about 15% lower than the estimated crystallinity degree of this polymer in the mats that do not contain NQ. This accounted for the detected amorphous halo of PLA in the XRD patterns of PEO/PLA/BW/NQ mats. For NQ, the DSC analyses revealed that a melting peak for the drug at ca. 180 ºC was recorded only for the PEO(50)/PLA(25)/BW(25)/NQ mat (i.e., for the mat having the highest content of PLA and BW). For the other PEO/PLA/BW/NQ mats, no melting peak for NQ was recorded, i.e., the drug is in the amorphous state in these materials.

The stress-strain curves registered for PEO/PLA/BW/NQ mats are shown in Supplementary Material, Figure S13a, and the values of the tensile strength and of Young’s modulus for the respective mats are presented in Supplementary Material, Figure S13b,c. As can be seen, increasing the PLA content in the fibrous materials did not significantly alter the tensile strength or Young’s modulus of the mats, and they were close to those determined for the neat PLA mat. This was attributed to the fact that PLA is in the amorphous state in PEO/PLA/BW/NQ fibrous materials, as evidenced by the XRD analyses (Supplementary Material, Figure S11a). In terms of the flexibility of PEO/PLA/BW/NQ fibrous materials, for the mats prepared at a weight ratio between the polymers and BW from 80/10/10 to 60/20/20, the presence of NQ did not alter this mechanical parameter, and again the ε value exceeded 200% and no break of the specimens was observed. For PEO(50)/PLA(25)/BW(25)/NQ, the detected elongation was ca. 70%, after which a break of specimens was detected. This behavior was attributed to the presence of the highest amount of the brittle BW, similar to the case of PEO(50)/PLA(25)/BW(25) mats.

### 3.3. Evaluation of the Effect of Electrospun Composite Materials from PEO/PLA/BW and PEO/PLA/BW/NQ on Contact with Pathogenic Microorganisms

The antibacterial activity of PEO/PLA/BW/NQ mats was assessed by determination of the inhibition zones around fibrous discs against the Gram-positive bacteria *S. aureus*, and the Gram-negative bacteria *E. coli* and *P. aeruginosa*, and their antifungal activity against *C. albicans*.

**Figure 10:**
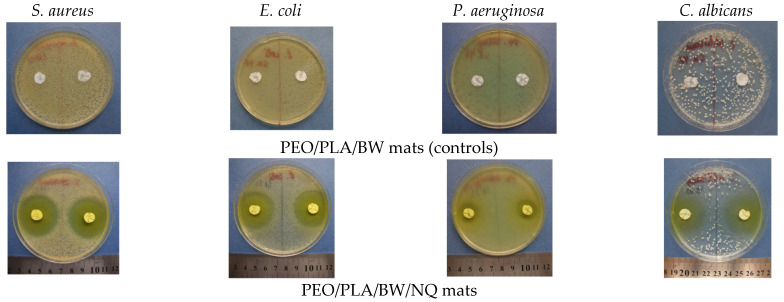
Digital images of the inhibition zones detected after a 24 h contact of PEO(50)/PLA(25)/BW(25)/NQ mats with *S. aureus*, *E. coli* or *P. aeruginosa* cells or after a 48 h contact with *C. albicans* (lower row). For comparison, images of Petri dishes with blank control PEO(50)/PLA(25)/BW(25) mats are presented on the upper row.

The minimum inhibitory concentration of NQ against *S. aureus*, *E. coli*, *P. aeruginosa* and *C. albicans* is 4.8 mg/L [48], 2.4 mg/L [48], 64 mg/L [49] and 8.0 mg/L [50], respectively. The mechanism of the antimicrobial activity of NQ is attributed to its ability to chelate divalent cations important for microbial growth [51,52]. Digital images of Petri dishes in which fibrous disks of PEO(50)/PLA(25)/BW(25) (control mats) and PEO(50)/PLA(25)/BW(25)/NQ were put into contact with the studied pathogenic microorganisms are presented in Figure 10. As can be seen, the control mats did not lead to inhibition of the development of the pathogens, i.e., PEO, PLA and BW exhibited no antimicrobial activity. The presence of the antimicrobial agent NQ in PEO(50)/PLA(25)/BW(25)/NQ mats led to the appearance of inhibition zones, indicating that these mats exhibited antibacterial and antifungal activities. As seen from Table 3, the composition of the fibrous carrier in terms of the ratio of polymers and BW did not alter the determined inhibition zone value against the studied pathogens.

**Figure 11:**
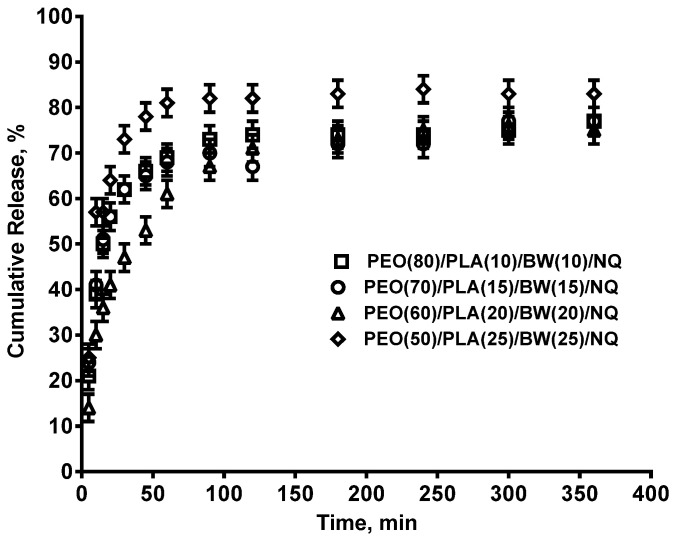
Release profiles of NQ from PEO/PLA/BW/NQ mats using a phosphate buffer solution of pH 7.4 and ionic strength of 0.1 at 37 ºC. Error bars correspond to ± SD values calculated based on three replicates for each time point.

This was attributed to the commensurate amount of NQ released over time, as was found by the study on the release of NQ from PEO/PLA/BW/NQ mats (Figure 11).

The smallest detected inhibition zone in the case of *P. aeruginosa* may be explained by that fact that it has the highest minimum inhibitory concentration of NQ, as compared to the other studied microorganisms.

## 4. Conclusions

As a continuation of our previous study [37], herein was reported the preparation of new composite fibrous materials from the PEO/PLA/BW system, employing single-spinneret electrospinning of homogeneous blend solutions. It was shown that core/double-sheath PEO/PLA/BW fibers were obtained. This was attributed to the difference in the molecular weights of the components of the blend solution, which resulted in different migration ability of the components to the surface of the spinning jet during the electrospinning process, and thus to the surface of the fibers. BW, as a complex mixture of low-molecular-weight substances, had the highest migration ability to the surface and, therefore, formed the outer fiber sheath. PLA, having intermediate molecular weight, showed lower mobility than BW but higher mobility than PEO, and thus PLA formed the inner sheath. PEO, as the component with the highest molar mass, formed the fiber core during the electrospinning process. In addition, the incompatibility between the partners and air hydrophobicity played roles in the formation of core/double-sheath fibers, as well. The possibility for PEO/PLA/BW to be used as carrier for bioactive agents was also demonstrated. It was shown that drug-loaded PEO/PLA/BW/NQ mats exerted antimicrobial activity, thus indicating that the new electrospun core-double sheath fibers could be used in medical applications, e.g., for wound-healing dressings.

## Figures and Tables

**Table 1:** Area of a segment from a fiber core and sheaths, as well as the sheath-to-core area ratio as estimated using the recorded TEM images.

Specimen	Core Area [µm^2^] ^(a)^	PLA Sheath [µm^2^] ^(a)^	BW Sheath [µm^2^] ^(a)^	Sheath-to-Core Area Ratio
PEO(80)/PLA(10)/BW(10)	1.13 ± 0.15	0.14 ± 0.01	0.13 ± 0.01	0.24
PEO(70)/PLA(15)/BW(15)	2.70 ± 0.26	0.60 ± 0.04	0.40 ± 0.03	0.37
PEO(60)/PLA(20)/BW(20)	0.94 ± 0.07	0.25 ± 0.02	0.21 ± 0.02	0.49
PEO(50)/PLA(25)/BW(25)	1.64 ± 0.16	0.33 ± 0.03	0.70 ± 0.04	0.63

^(a)^ The estimation was performed by the Image J program using TEM micrographs of the specimens at a magnification of ×10,000 and at a constant fiber length of 1.5 µm, applying a methodology described in detail in [37].

**Table 2:** Melting temperature and enthalpy of PLA and PEO/BW, registered by DSC. PLA crystallinity degree in the composite fibrous materials.

Specimen	T_m_^PLA^ [ºC]	ΔH_m_^PLA^ [J/g]	*χ*_c_^PLA^ [%] ^(a)^	T_m_^PEO/BW^ [ºC]	ΔH_m_^PEO/BW^ [J/g]
PEO(80)/PLA(10)/BW(10)	166	4.3	46	65	113
PEO(70)/PLA(15)/BW(15)	166	6.4	46	65	109
PEO(60)/PLA(20)/BW(20)	166	8.6	46	65	103
PEO(50)/PLA(25)/BW(25)	166	10.8	46	65	102

^(a)^ PLA crystallinity degree in the composite fibrous materials was calculated using the following equation: *χ*_c_^PLA^, % = [ΔH_m_^PLA^]/(ΔH_m_^PLA,0^ × W^PLA^)] × 100, where ΔH_m_^PLA,0^ = 93.0 J/g [40], and W^PLA^ was the weight fraction of PLA in the composite fibrous materials.

**Table 3:** Inhibition zones determined after a 24 h contact of PEO/BW/PLA/NQ mats with *S. aureus*, *E. coli*, *P. aeruginosa* or after a 48 h contact with *C. albicans*.

Specimen	Inhibition Zone [cm]			
	*S. aureus*	*E. coli*	*P. aeruginosa*	*C. albicans*
PEO(70)/PLA(15)/BW(15)	3.3 ± 0.1	3.5 ± 0.2	2.2 ± 0.1	3.3 ± 0.3
PEO(50)/PLA(25)/BW(25)	4.2 ± 0.2	3.5 ± 0.2	1.9 ± 0.1	3.2 ± 0.2

## Data Availability

Not applicable.

## Supplementary Materials

The following supporting information can be downloaded at: https://www.mdpi.com/article/10.3390/polym14225036/s1, Figure S1: Dependence of the mean diameters of the fibers prepared at different PEO/PLA/BW ratios on PLA content in the spinning solution. Inset figure: dependence of the dynamic viscosity of PEO/PLA/BW solutions on PLA content; Figure S2: Dependence of the weight loss of PEO(80)/PLA(10)/BW(10) (1), PEO(70)/PLA(15)/BW(15) (2), PEO(60)/PLA(20)/BW(20) (3) and PEO(50)/PLA(25)/BW(25) (4) on mat composition after a 24 h stay of the fibrous materials in hexane. For comparison, the theoretical weight loss as calculated assuming that all of the BW has been removed after the stay of the mats in hexane is also presented; Figure S3: SEM (a) and TEM (b,c) micrographs of PEO(60)/PLA(20)/BW(20) fibers after a subsequent stay of the mat in hexane and distilled water; Figure S4: Juxtaposition of the FTIR spectra of BW pellets, a PLA mat, a PEO mat and a PEO(60)/PLA(20)/BW(20) mat before and after treatment with hexane alone (24 h) or after subsequent treatment with hexane (24 h) and water (48 h); Figure S5: SEM micrographs of PEO/PLA/BW mats after a 24 or 48 h stay in distilled water. Magnification: ×500; Figure S6: XRD patterns of PEO/PLA/BW mats after a 24 h stay in hexane; Figure S7: Digital images of PEO/PLA/BW fibrous specimens before tensile tests (a), and after the performance of tensile tests on PEO(60)/PLA(20)/BW(20) (b) and PEO(50)/PLA(25)/BW(25) (c) mats; Figure S8: SEM micrographs of fibrous materials from PEO(80)/PLA(10)/BW(10)/NQ (a) and PEO(50)/PLA(25)/BW(25)/NQ (b). Magnification: ×1000; Figure S9: A TEM micrograph of PEO(50)/PLA(25)/BW(25)/NQ fiber. Magnification: ×10,000; Figure S10: Digital images of water droplets deposited on a PEO(50)/PLA(25)/BW(25)/NQ mat; Figure S11: XRD patterns of PEO/PLA/BW/NQ fibrous materials prepared at different weight ratios between the polymers and BW, and equal NQ content 10% of weight with respect to the total weight of the solids (a). For comparison, XRD patterns of NQ powder are presented, as well (b); Figure S12: DSC thermograms (first heating run) of fibrous materials: PEO(80)/PLA(10)/BW(10)/NQ (dark blue line), PEO(70)/PLA(15)/BW(15)/NQ (red line), PEO(60)/PLA(20)/BW(20)/NQ (green line) and PEO(50)/PLA(25)/BW(25)/NQ (light blue line). Registered using differential scanning calorimeter Perkin Elmer DSC 8500; Figure S13: Stress-strain curves for PEO/PLA/BW/NQ fibrous materials (a). Tensile strength (b) and Young’s modulus (c) of PEO(80)/PLA(10)/BW(10)/NQ (1), PEO(70)/PLA(15)/BW(15)/NQ (2), PEO(60)/PLA(20)/BW(20)/NQ (3) and PEO(50)/PLA(25)/BW(25)/NQ (4) mats.
