# Supplementary material for: Core/Double-Sheath Composite Fibers from Poly(ethylene oxide), Poly(L-lactide) and Beeswax by Single-Spinneret Electrospinning
Source: Polymers (Basel). 2022 Nov 21;14(22):5036. doi: 10.3390/polym14225036 (PMC9699041; doi:10.3390/polym14225036)
Supplement: Supplementary file 1 [file polymers-14-05036-s001.zip › polymers-2031316-supplementary.pdf]

Supplementary Material

Core/Double-Sheath Composite Fibers from Poly(ethylene oxide), Poly(L-lactide) and Beeswax by Single-Spinneret Electrospinning

Selin Kyuchyuk, Dilyana Paneva*, Nevena Manolova*, Iliya Rashkov, Daniela Karashanova, Nadya Markova

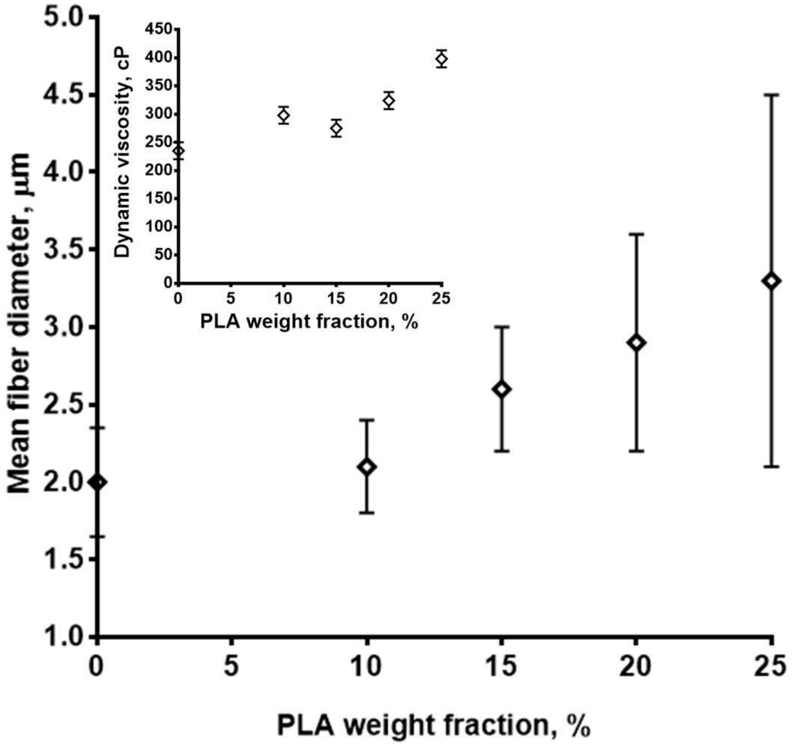

Figure S1. Dependence of the mean diameters of the fibers prepared at different PEO/PLA/BW ratios on PLA content in the spinning solution. Inset figure: dependence of the dynamic viscosity of PEO/PLA/BW solutions on PLA content.

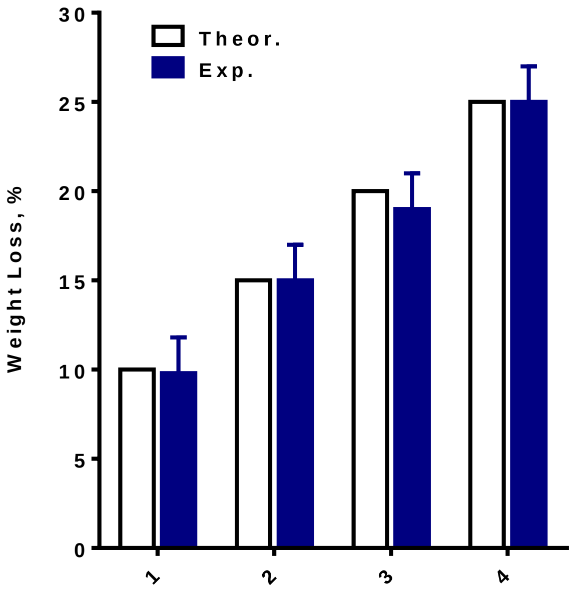

Figure S2.
Dependence of the weight loss of PEO(80)/PLA(10)/BW(10) (1), PEO(70)/PLA(15)/BW(15) (2), PEO(60)/PLA(20)/BW(20) (3) and PEO(50)/PLA(25)/BW(25) (4) on mats composition after a 24-h stay of the fibrous materials in hexane. For comparison, the theoretical weight loss as calculated assuming that the whole BW amount has been removed after the stay of the mats in hexane is also presented.

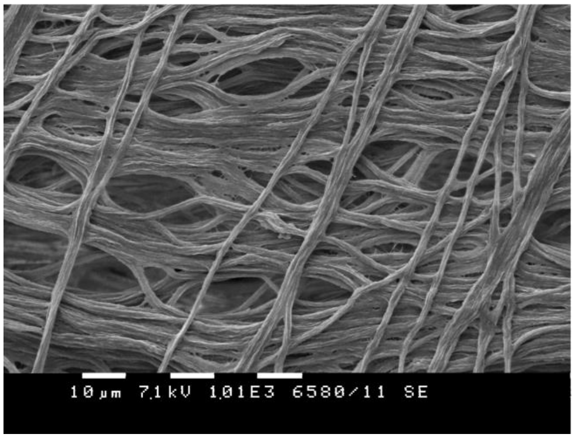

a, × 1 000

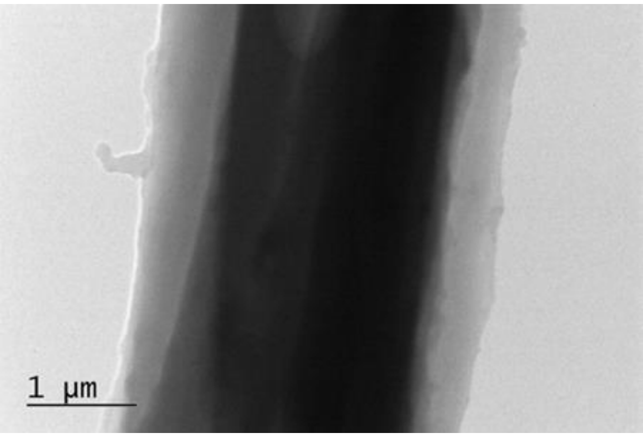

b, × 5 000

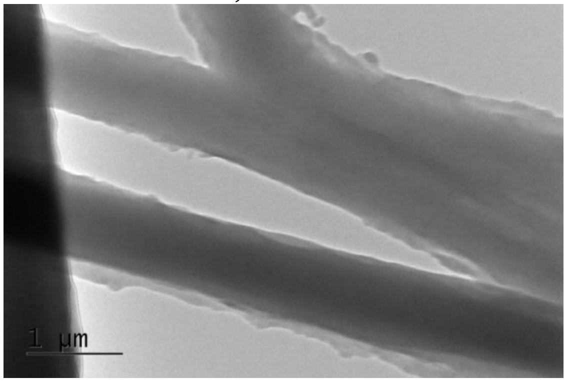

c, × 5 000

Figure S3.
SEM (a) and TEM (b, c) micrographs of PEO(60)/PLA(20)/BW(20) fibers after a subsequent stay of the mat in hexane and distilled water.

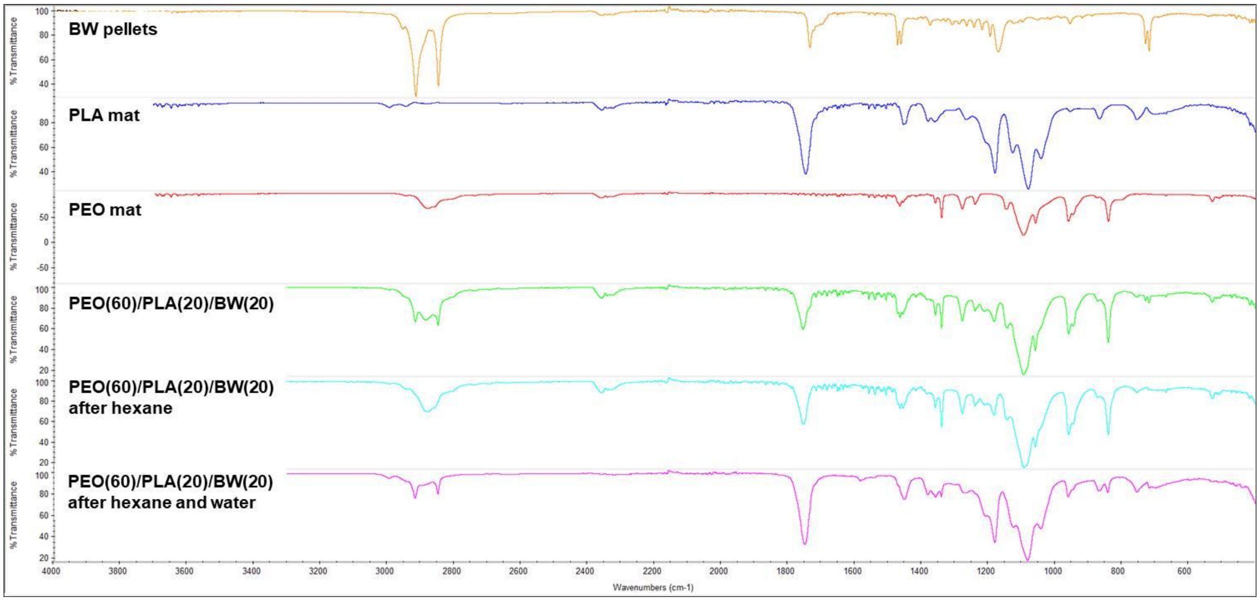

Figure S4. Juxtaposition of the FTIR spectra of BW pellets, PLA mat, PEO mat and PEO(60)/PLA(20)/BW(20) mat before and after treatment with hexane alone (24 h) or after subsequent treatment with hexane (24 h) and water (48 h).

PEO(80)/PLA(10)/BW(10)

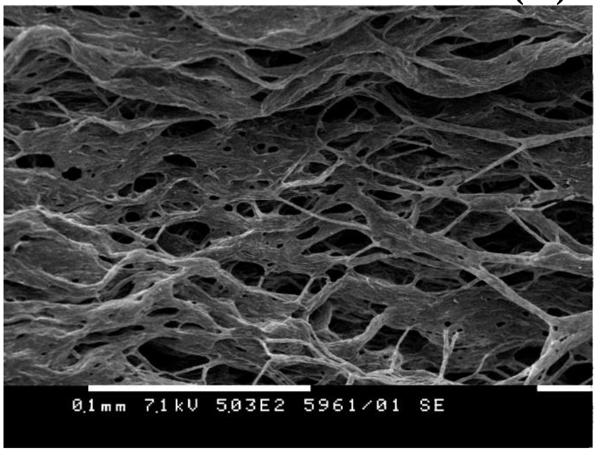

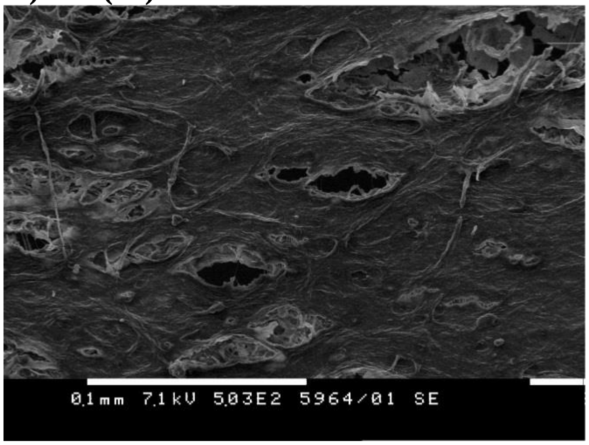

24 h **48 h**

PEO(50)/PLA(25)/BW(25)

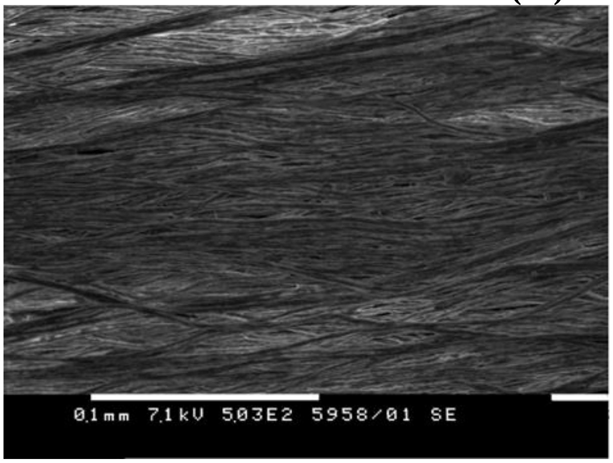

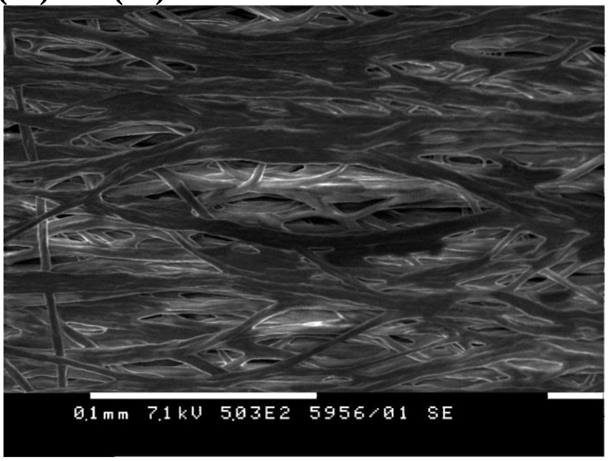

24 h **48 h**

Figure S5. SEM micrographs of PEO/PLA/BW mats after a 24- or 48-h stay in distilled water. Magnification: × 500.

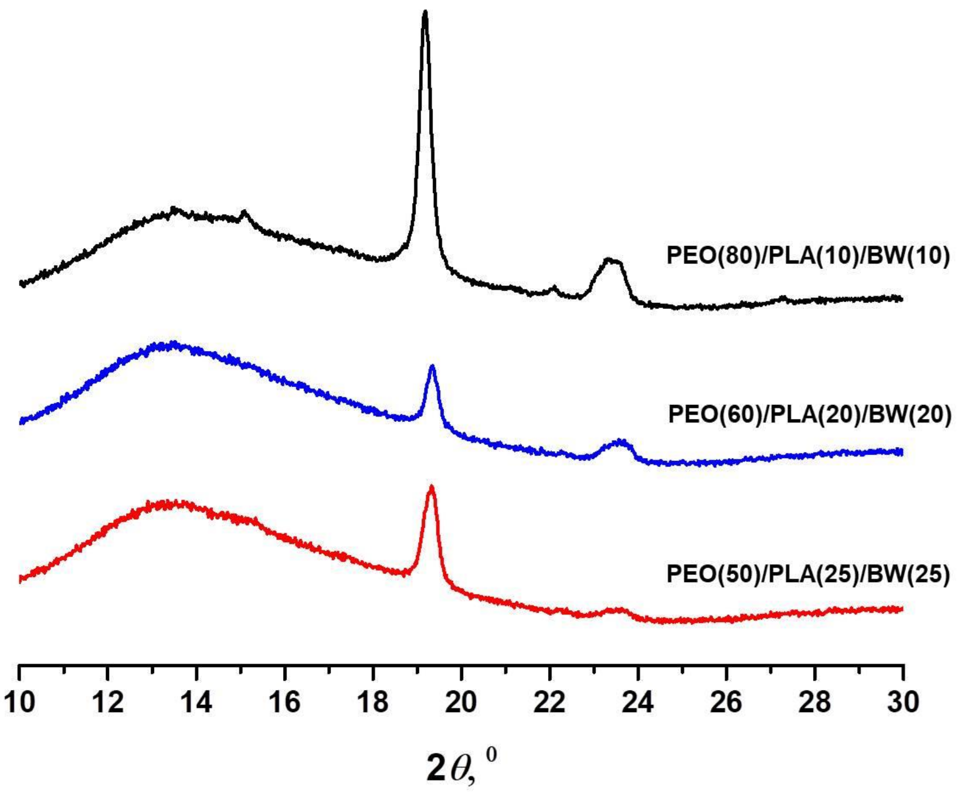

Figure S6. XRD patterns of PEO/PLA/BW mats after a 24-h stay in hexane.

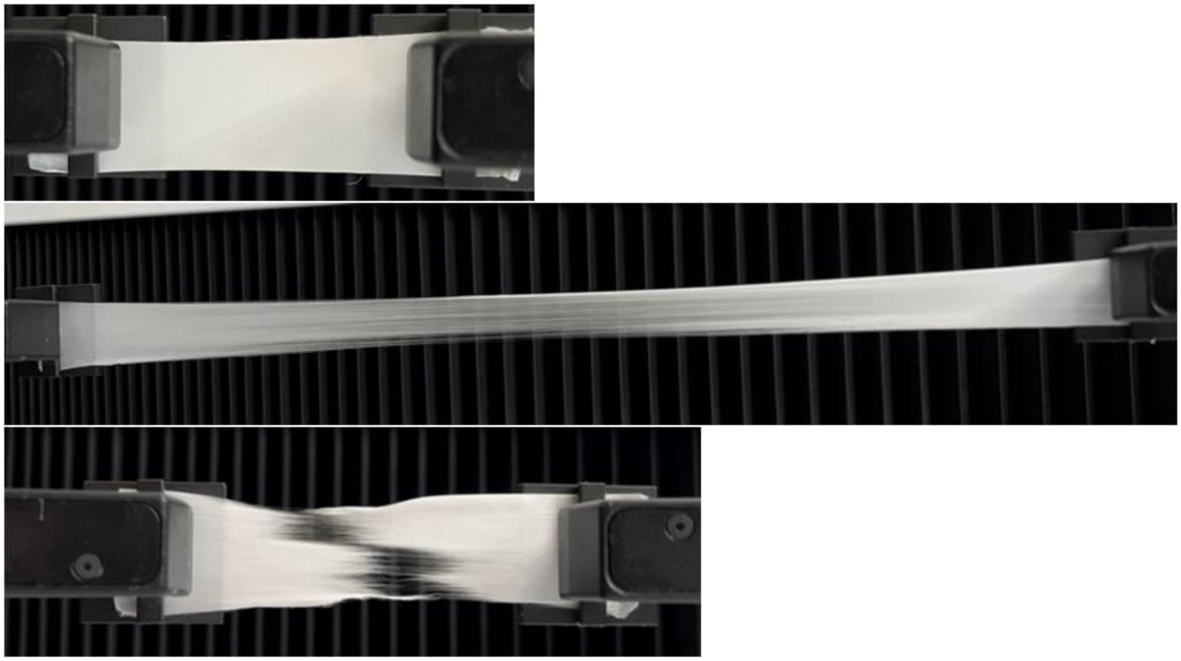

a

b

c

Figure S7. Digital images of PEO/PLA/BW fibrous specimens before tensile tests (a), and after the performance of tensile tests of PEO(60)/PLA(20)/BW(20) (b) and PEO(50)/PLA(25)/BW(25) (c) mats.

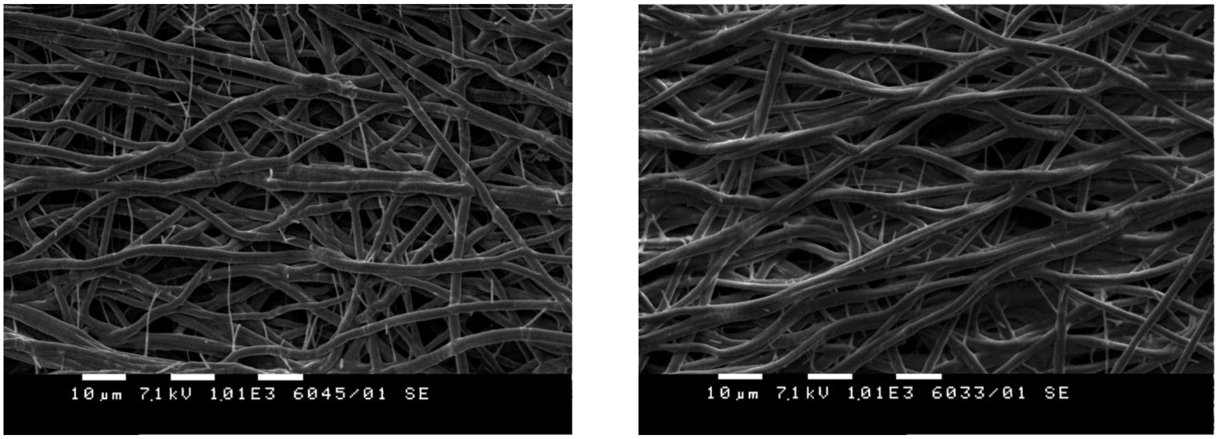

a 2.3 ± 0.374 µm

b 2.3 ± 0.236 µm

Figure S8. SEM micrographs of fibrous materials from PEO(80)/PLA(10)/BW(10)/NQ (a) and PEO(50)/PLA(25)/BW(25)/NQ (b). Magnification: × 1 000.

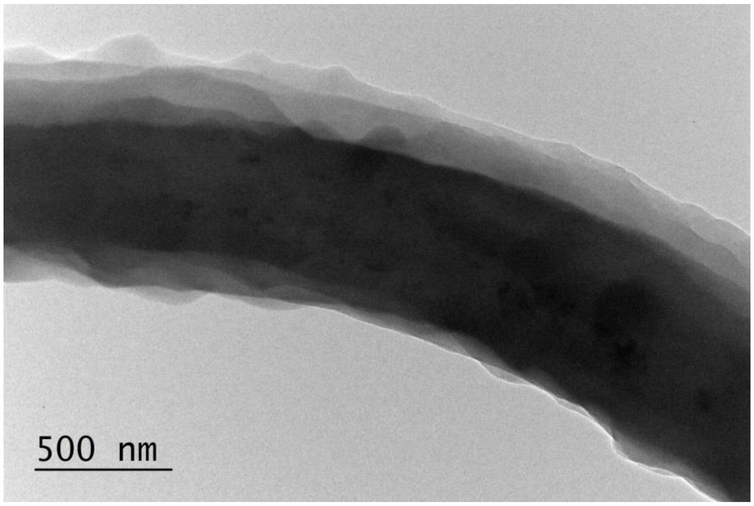

Figure S9. A TEM micrograph of PEO(50)/PLA(25)/BW(25)/NQ fiber. Magnification: × 10 000.

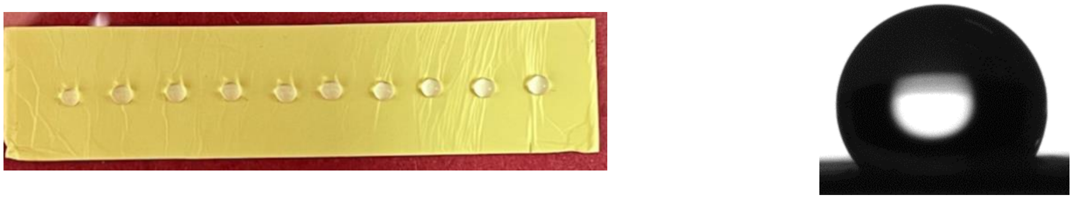

Figure S10. Digital images of water droplets deposited on PEO(50)/PLA(25)/BW(25)/NQ mat.

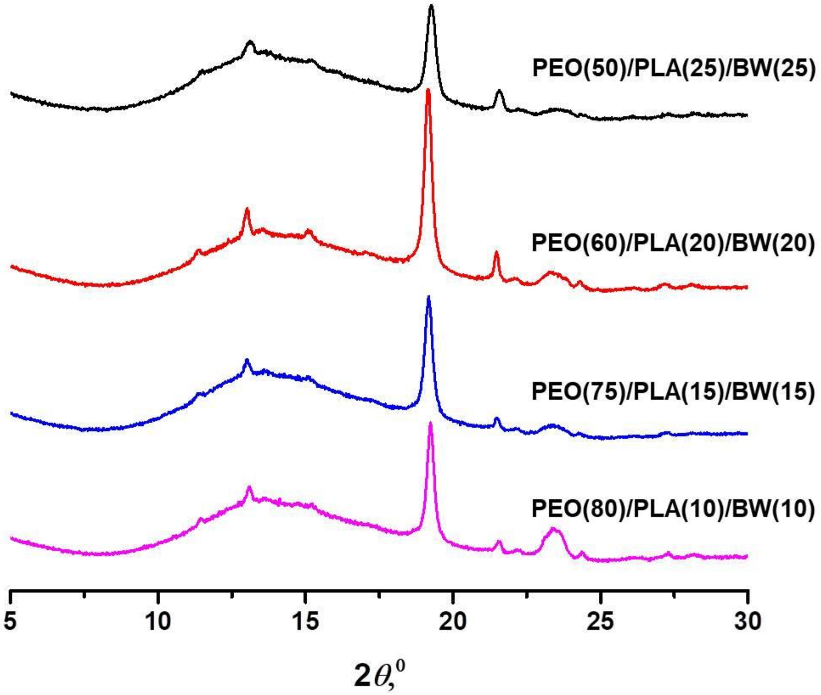

a

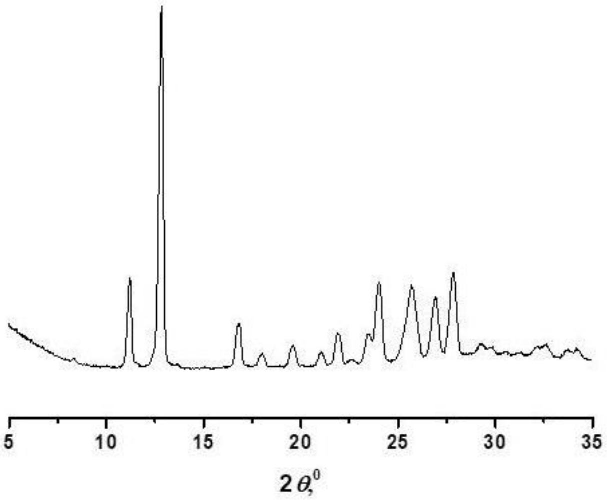

b

Figure S11. XRD patterns of PEO/PLA/BW/NQ fibrous materials prepared at different weight ratio between the polymers and BW and equal NQ content – 10 wt.% in respect to the total weight of the solids (a). For comparison, XRD patterns of NQ powder is presented, as well (b).

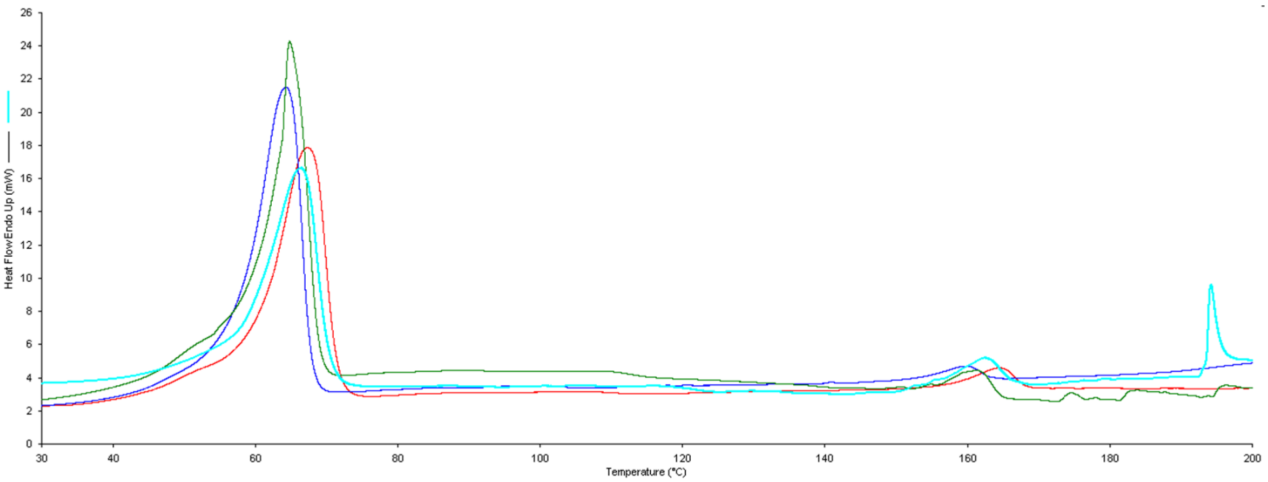

Figure S12. DSC thermograms (first heating run) of fibrous materials from: PEO(80)/PLA(10)/BW(10)/NQ (dark blue line); PEO(70)/PLA(15)/BW(15)/NQ (red line); PEO(60)/PLA(20)/BW(20)/NQ (green line), and PEO(50)/PLA(25)/BW(25)/NQ (light blue line); registered using differential scanning calorimeter Perkin Elmer DSC 8500.

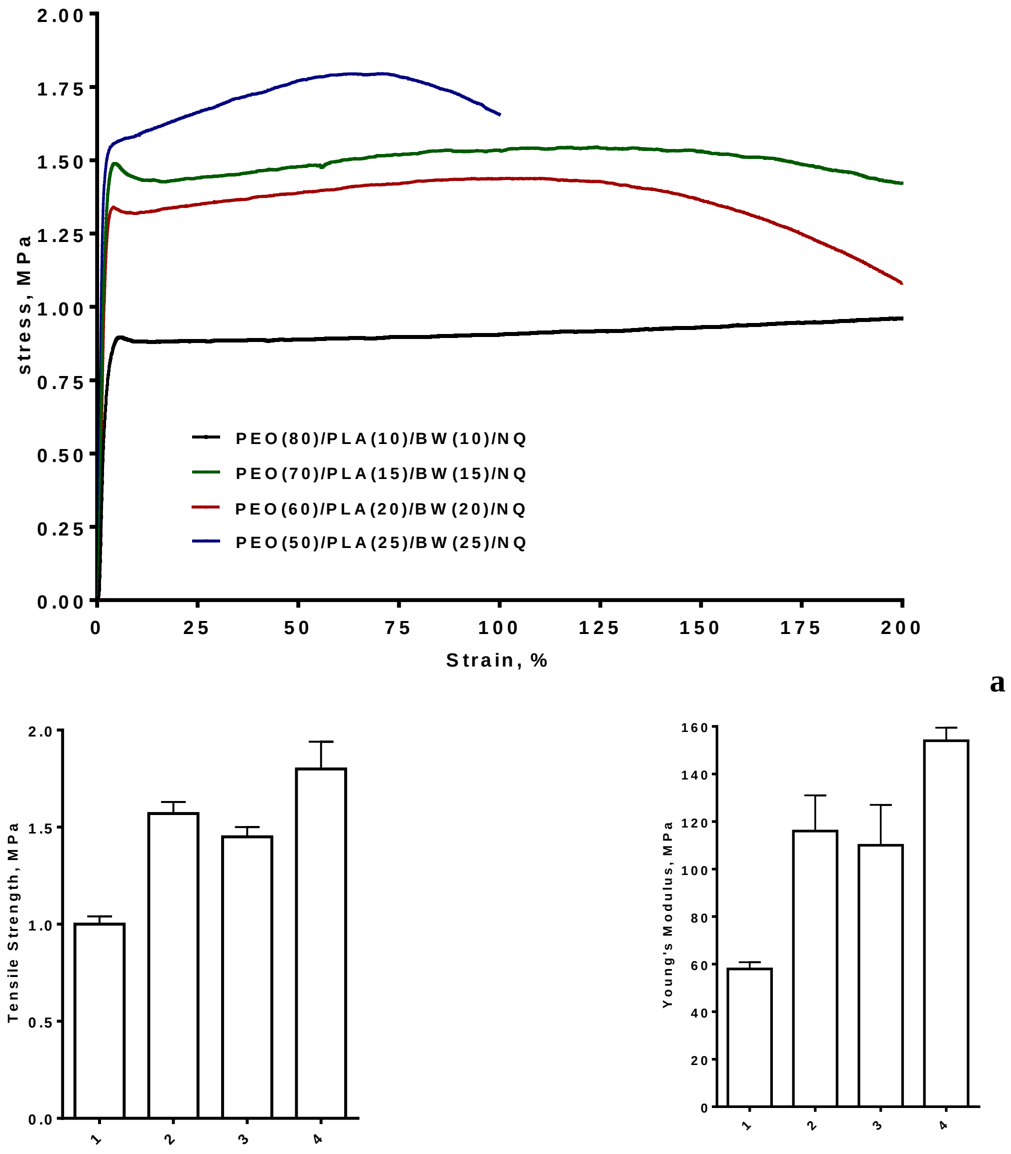

Figure S13. Stress-strain curves for PEO/PLA/BW/NQ fibrous materials (a). Tensile strength (b) and Young's modulus (c) of PEO(80)/PLA(10)/BW(10)/NQ (1); PEO(70)/PLA(15)/BW(15)/NQ (2); PEO(60)/PLA(20)/ BW(20)/NQ (3) and PEO(50)/PLA(25)/BW(25)/NQ (4) mats.
